# Valsartan Improves Endothelial Dysfunction in Hypertension: A Randomized, Double-Blind Study

**DOI:** 10.1111/j.1755-5922.2009.00085.x

**Published:** 2009

**Authors:** Nikolaos Tzemos, Pitt O Lim, Thomas M MacDonald

**Affiliations:** Hypertension Research Centre, Division of Medicine and Therapeutics, University of Dundee, Ninewells Hospital and Medical SchoolDundee, UK

**Keywords:** Amlodipine, Blood pressure, Endothelium, Nitric oxide, Valsartan

## Abstract

Endothelial dysfunction can predict cardiac outcomes in hypertension and reversing this abnormality has become an attractive therapeutic objective. We tested the hypothesis that blocking the angiotensin type 1 (AT_1_) receptor with valsartan in comparison with amlodipine would lead to an improvement in forearm resistance artery endothelial dysfunction. In total, 25 hypertensive subjects (mean age 60 years, SD 8) with a mean daytime ambulatory blood pressure (BP) of 154 (10)/97 (6) mmHg were randomized following a 3-week placebo run-in period to a double-blind, crossover trial of 16-week treatment periods with either valsartan or amlodipine, separated by a 3-week washout period. Intra-arterial infusions of acetylcholine (ACh) and N^G^-monomethyl-L-arginine (L-NMMA) were used to assess stimulated and basal endothelium-dependent nitric oxide (NO) release, respectively. Coinfusion of ACh and L-NMMA was employed to investigate the existence of an NO-independent vasodilatory pathway. Valsartan and amlodipine each lowered the clinical BP to the same extent (139 [7]/87 [6] and 139 [11]/89 [4] mmHg, respectively). The vasodilatory response to ACh was significantly increased with valsartan (maximal percentage change in forearm blood flow (max. ΔFBF%) 301 [47] vs. 185 [34], mean [SEM]; *P* < 0.05) as compared with placebo, but remained unchanged with amlodipine. Both valsartan and amlodipine similarly increased the vasoconstrictive response to L-NMMA (max. ΔFBF%–43 [5], −42 [5], respectively, vs. –26 [3] baseline; *P* < 0.001). The vasodilatory response after coinfusion of ACh and L-NMMA was significantly (*P* < 0.05) enhanced only with valsartan. Valsartan reserved peripheral endothelial dysfunction through both NO-dependent and -independent pathways, while for the same degree of BP control, amlodipine had only a partial effect on NO bioactivity.

## Introduction

An impairment of nitric oxide (NO) bioactivity or endothelial dysfunction is an early feature found in small resistance arteries of various vascular diseases, including hypertension, and is often present before any structural abnormality is evident [1,2]. Most, but not all, reports have confirmed the presence of endothelial dysfunction in essential and secondary hypertension [1–3] Although the mechanisms of NO impairment in hypertension are likely to be complex and heterogeneous, they are also desirable therapeutic targets since the presence of endothelial dysfunction conveys an adverse cardiac prognosis [4,5].

Recent research has focused on angiotensin II as a mediator of endothelial dysfunction in various cardiovascular disorders [6,7]. Angiotensin II via the angiotensin type 1 (AT_1_) receptor causes arteriolar vasoconstriction and remodeling, superoxide anion production, renal sodium reabsorption, aldosterone secretion, and endothelin release, leading to increased vascular resistance and promoting atherosclerosis [8,9]. Thus, AT_1_ receptor activation limits NO bioactivity both by reducing NO release and by increasing NO inactivation. We investigated the effects of AT_1_ receptor blockade, with valsartan, on blood pressure (BP) and NO bioactivity in patients with essential hypertension and compared this with calcium channel blockade with amlodipine.

## Methods

### Subjects

Twenty-five subjects (17 males and 8 postmenopausal females) with a mean age of 60 years (SD 8) and long-standing, treated, uncomplicated essential hypertension and without electrocardiographic or echocardiographic evidence of left ventricular hypertrophy were recruited from those attending the Tayside hypertension clinic. The secondary causes of hypertension were excluded by history, physical examination, and biochemical and imaging studies, where clinically indicated. Patients with a history of coronary artery disease, diabetes, hyperlipidemia (total cholesterol >5.0 mmol/L), renal impairment, or other vascular diseases were also excluded. Twenty-five healthy volunteers matched for sex and age recruited through advertisement within our institution constituted the control comparison group. The study was approved by the Tayside ethics committee.

### Study Protocol

This was a randomized, double-blind, placebo-controlled crossover study. The patients were randomized to receive either valsartan or amlodipine in a crossover design, each for 16 weeks, with a 3-week washout period between treatments. After randomization, all antihypertensive drugs (Table 1) were withdrawn, and the subjects entered a 3-week placebo run-in period. None of the patients, either before or during the study, were taking any medication known to affect the vascular endothelium. At the end of the 3-week placebo run-in period, all subjects underwent a 24-h ambulatory blood pressure monitoring (ABPM) (Table 2). Also, vascular endothelial function assessment and blood sampling took place at the end of the same period (baseline). The subjects were then randomized to 16-week treatment periods of either valsartan 80 mg or amlodipine 5 mg. The subjects returned subsequently to the laboratory after 4 weeks in each active treatment period. If their BP was >140/90 mmHg, then the study medication was uptitrated to 160 mg for valsartan or 10 mg for amlodipine. Finally, at the end of each active treatment, a further 3-week placebo period (washout) was implemented prior to crossing over to the alternative drug.

**Table 2:** Hemodynamic data during the study

Variable	Baseline (placebo)	Amlodipine	Valsartan
24-h SBP, mmHg	155 (3)	135 (3)*	136 (4)*
24-h DBP, mmHg	92 (3)	84 (3)*	83 (2)*
Daytime SBP, mmHg	151 (11)	138 (4)*	139 (4)*
Daytime DBP, mmHg	97(3)	87 (4)*	88 (3)*
Clinical SBP, mmHg	162 (13)	139 (11)*	139 (7)*
Clinical DBP, mmHg	103 (8)	87 (6)*	89 (4)*
Heart rate, beats/min	75 (9)	76 (12)	79 (12)
Absolute basal FBF	3 (0.2)	3.1 (0.3)	3.1(0.2)

24-h indicates daytime and nighttime ambulatory blood pressure monitoring, respectively, for systolic blood pressure (SBP) and diastolic blood pressure (DBP). FBF indicates forearm blood flow expressed in mL/min/100 mL forearm volume.

Values are expressed as mean (SD).

* *P* < 0.05 versus baseline (placebo).

**Table 1:** Previous antihypertensive treatment of the study population

Number (percentage) of patients	Previous antihypertensive therapy
14 (56)	βeta receptor blocker (atenolol)
6 (24)	Calcium channel blocker (amlodipine)
5 (20)	ACE-I (lisinopril)
6 (24)	Combination with thiazide diuretic

ACE-I indicates angiotensin-converting enzyme inhibitor.

Clinical BP was measured (a mean of three measurements) at the beginning of each visit after 10-min rest in seated position using a semiautomatic oscillometric monitor (OMRON 705CP; Matsusaka, Matsusaka-City, Japan). ABPM was recorded using SpaceLabs model 90207 recorders (Redmond, Washington, DC, USA). Recordings were taken every 15 min during the daytime (8.00 a.m. to 10.00 p.m) and every 30 min during the nighttime (10.00 p.m. to 8.00 a.m.). During ABPM, the subjects were asked to continue their normal daily life activities.

### Vascular Studies

Vascular studies took place at the end of the 3-week placebo run-in period (baseline) and at the end of each 16-week active treatment; thus, each subject underwent, in total, three endothelial function assessments. All vascular studies were conducted by the same operator (N.T.) after an overnight fast and in a quiet, temperature-controlled laboratory (24 ± 0.5°C) with dimmed lights. Alcohol and caffeine-containing beverages were avoided for at least 24 h before the study day. Following a supine rest of 30 min, the nondominant brachial artery was cannulated with a 27-gauge steel needle mounted onto a 16-gauge polyethylene epidural catheter under local anesthesia with 1% lidocaine. Forearm blood flow (FBF) was measured simultaneously in both arms by strain-gauge venous occlusion plethysmography, as previously described [10]. BP and heart rate were noninvasively (HEM-705CP; OMRON, Matsusaka-City, Japan) recorded in the noninfused (control) arm before each infusion.

## Hemodynamic Measurements and Drug Infusions

FBF was measured during the last 2 min after each infusion period and expressed as mL/min/100 mL forearm volume according to the Whitney method [11]. Resting baseline FBF values were obtained at least 30 min after needle placement to ensure that the blood flow in the cannulated arm had stabilized. After resting baseline FBF measurements, each study subject received intra-arterial infusions of incremental doses of acetylcholine (ACh, Miochol; CIBAVision, Southampton, UK), sodium nitroprusside (SNP; David Bull Laboratories, Warwick, UK), and N^G^-monomethyl-L-arginine (L-NMMA; Clinalfa, Läufelfingem, Switzerland). The muscarinic agonist ACh was used to assess endothelium-dependent vasodilatation (stimulated NO release), while SNP, an exogenous source of NO, was used to assess endothelium-independent vasodilatation. Cumulative dose response curves were constructed after infusions of ACh at 25, 50, and 100 nmol/min and SNP at 4.2, 12.6, and 37.8 nmol/min, each incremental dose for 5 min. The endothelial-dependent vasoconstriction was assessed using the competitive nitric oxide synthase (NOS) antagonist L-NMMA infused at 1, 2, and 4 μmol/min, again, each for 5 min. After each agent, care was taken for FBF to reach the baseline values, generally at least after 30 min. To assess the presence of NO-independent pathway in achieving resistance artery vasodilation, L-NMMA infusion was kept constant at 4 μmol/min for further 10 min. Then, ACh at 50 and 100 mol/min, each for 5 min, was coinfused with L-NMMA and respective dose–response curves were constructed. The order of the vasoactive drugs infused was identical in all the study visits. Drugs, saline, and 5% dextrose were infused at flow rates of 1 mL/min by means of a constant-rate infusion pump (Braun, Sheffield, UK). All studies were performed by the same operator (N.T.), blinded to the other measurements.

### Statistical Analysis

Five recordings at each infusion step were measured for both the infused and the control arms. Because BP and baseline FBFs did not vary significantly during the visits, FBF ratio between the infused and the control arm in response to drugs was expressed as a percentage of the ratio measured during the control period (percentage change in FBF, ΔFBF%[mean ± SEM]). From previous studies, the sample size was estimated to have a power of 90% to detect a cumulative ΔFBF% difference between treatments of 100%. Clinical characteristics between clinic visits were compared by Student's paired *t*-test, while FBF measurements for individual treatments were compared between the treatments by a two-way ANOVA with repeated measures, with correction for multiple comparisons for within-group effects. The Wilcoxon rank-sum test was used when variables were not normally distributed. A two-tailed *P* < 0.05 was considered significant. Sensitivity and reproducibility of the methods performed in our laboratory have been previously reported [10].

## Results

### Clinical and Biochemical Characteristics

Daytime ABPM (Table 2) at the end of the 3-week placebo run-in period confirmed mild-to-moderate essential hypertension [12]. There were no significant differences in baseline plasma electrolytes or cholesterol levels between the three study periods (Table 3). A significant reduction in plasma aldosterone was observed only with valsartan treatment compared with baseline (placebo) (Table 2). Ninety percent of all patients in each treatment group required their antihypertensive therapy titrated to a double dose.

**Table 3:** Biochemical parameters during the study

Variable	Baseline (placebo)	Amlodipine	Valsartan
Serum potassium, mmol/L	4.1 (0.3)	4.0 (0.2)	4.2 (0.3)
Serum creatinine, μmol/L	95 (10)	94 (12)	93 (10)
Plasma urate, mmol/L	0.34 (0.3)	0.30 (0.3)	0.34 (0.3)
Plasma glucose, mmol/L	4.9 (0.3)	5.1 (0.7)	4.8 (0.6)
Total cholesterol, mmol/L	4.8 (0.8)	4.9 (0.6)	4.8 (0.5)
Plasma aldosterone, pg/mL	136 (20)	126 (22)	75 (20)*

Values are expressed as mean (SD).

* *P* < 0.05 versus baseline (placebo).

At the end of the each 16-week treatment period, both valsartan and amlodipine reduced systolic and diastolic BP to the same extent compared with placebo (baseline) (Table 2).

### Baseline FBF Responses

**Figure 1:**
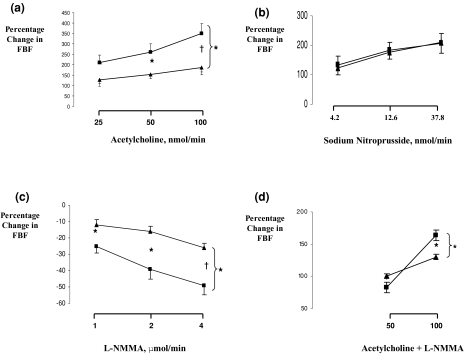
Percentage changes in forearm blood flow (FBF) ratio (infused/noninfused) from baseline preceding each drug infusion for three dose levels of (**A**) acetycholine, (**B**) sodium nitroprusside, and (**C**) L-NMMA and (**D**) coinfusion of acetylcholine and L-NMMA in normotensive control group (▪) and hypertensive patients (▴), respectively. Values are mean (SEM). *P < 0.05, ^†^*P* < 0.001 for differences between the treatments.

Patients with hypertension had a blunted vasodilatory response to ACh as compared with normotensive patients (*P* < 0.01 for cumulative dose), while the vasodilatory response to SNP, a surrogate of endothelial-independent vasodilation, was not different between the two groups (Figure 1). Similarly, the vasoconstrictive response was significantly blunted in hypertensive patients (*P* < 0.05 for cumulative dose; Figure 1). Coinfusion of L-NMMA and ACh produced significantly attenuated vasodilation in the normotensive group and, to lesser extent, in the hypertensive group (*P* < 0.05 for cumulative maximum ΔFBF%; Figure 1).

### FBF Responses during Antihypertensive Therapy

**Figure 2:**
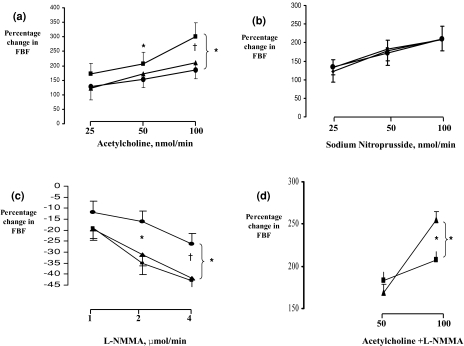
Percentage changes in forearm blood flow (FBF) ratio (infused/noninfused) from baseline preceding each drug infusion for three dose levels of (**A**) acetycholine, (**B**) sodium nitroprusside, and (**C**) L-NMMA and (**D**) coinfusion of acetylcholine and L-NMMA after placebo (•), valsartan (▪), and amlodipine (▴) treatment periods, respectively. Values are mean (SEM). *P < 0.05, ^†^*P* < 0.001 for differences between the treatments.

Valsartan produced a significant dose-dependent increase in forearm vasodilatory reponse to ACh as compared with baseline (maximum ΔFBF% 301 [47] vs. 185 [34], mean [SEM]: *P* < 0.01 for the difference between the whole-dose–response curves; Figure 2). In comparison, amlodipine had no significant effect on ACh vasodilatory response (ΔFBF% 210 [54] vs. 185 [34], *P*= 0.63; Figure 2). Neither valsartan nor amlodipine affected the vascular response to SNP significantly, suggesting that the vasodilatory NO-independent pathway remained unaffected (Figure 2). The vasoconstrictive response to exogenous NOS inhibitor L-NMMA was similarly and significantly enhanced with both valsartan and amlodipine compared with baseline (maximum ΔFBF%–43 [5], −42 [5], respectively, vs. –26 [3]; *P* < 0.05; Figure 2). Finally, during valsartan therapy, coinfusion of ACh and L-NMMA led to a significant increase in maximal ΔFBF% and thus to vasodilation in comparison to amlodipine therapy (Figure 2). Since pharmacologically evoked NO release was maximally exploited during coinfusion of ACh and L-NMMA, we speculate that the significant difference in FBF between the valsartan and the amplodipine periods was secondary to the existence of a NO-independent vasodilatory pathway.

## Discussion

Our study has two novel findings. First, we have shown that after a sufficiently long period of antihypertensive therapy with either valsartan or amlodipine and for the same BP control, only valsartan improved both stimulated and basal NO release. Second, and for the first time, we have shown that valsartan modulated forearm resistance artery endothelial function through the NO-independent mechanism.

### Endothelial Dysfunction in Hypertension and Renin–Angiotensin–Aldosterone System (RAAS)

Hypertension is a disease in which the systemic arteries are both structurally and functionally abnormal [13]. Apart from structural changes, the arterial wall also exhibits functional abnormalities such as impaired NO bioactivity or endothelial dysfunction [2]. Angiotensin II acting via the AT_1_ receptor has a direct vasocontrictive effect [14]: it promotes endothelin (ET-1) synthesis and release from the endothelial cells, thus causing vasoconstriction, and reduces NO release, thus increasing peripheral vascular resistance [15]. In addition, angiotensin II enhances NO inactivation via an increase in oxidative stress through NADP/NADPH activation [9]. It is conceivable that AT_1_ receptor blockade could prevent these adverse effects of angiotensin II on endothelial NO bioavailability.

### AT_1_ Receptor Blockade and Endothelial Function in Hypertensive Patients

Our study confirms previous reports of the beneficial effect of AT_1_ receptor blockade on vascular endothelium in those patients with documented atherosclerosis and also in hypertensive patients [7,16]. Schiffrin and colleagues [16] have shown that long-term treatment with another AT_1_ receptor blocker, losartan, improved both structural and functional properties of small resistance arteries in patients with essential hypertension. More recently, Klingbeil et al. [17] have shown in a carefully conducted double-blind, placebo controlled study that 6 weeks of oral valsartan therapy also favorably affected basal NO but had no effect on stimulated NO release. It is possible that a short duration of valsartan therapy (6 weeks compared with 16 weeks) might have been responsible for the lack of improvement in ACh-evoked vascular response. However, not all studies involving AT_1_ receptor blockers have had the same impact on NO bioactivity. Ghiadoni and colleagues [6] have reported that AT_1_ receptor blockade with candesartan improved basal and stimulated NO release in essential hypertensive patients. However, the improvement in stimulated NO release was associated with a concomitant enhancement of the SNP vasodilatory response; thus, improvement of both functional and structural properties of the arterial wall was evident. The patients involved in their study were highly selected and were mainly untreated (and probably had a shorter duration of hypertension), which may account for the disparity between their findings and ours. Also, we speculate that the different pharmacokinetic and pharmacodymamic properties of agents within the same class might have a profound difference on their biological effect, as in the case of angiotensin-converting enzyme (ACE) inhibitors [18].

### Mechanism(s) of Endothelial Dysfunction Reversal

The finding that valsartan therapy produced significant vasodilation during coinfusion of ACh and L-NMMA is intriguing. Additional vasodilation following coinfusion of ACh and L-NMMA suggests the existence of a non-NO pathway responsible for this effect. Although, not readily obvious from our study, it is possible that valsartan might have modulated peripheral endothelial function through a cyclo-oxygenase mechanism [19] and/or through the release of the elusive endothelium-derived hyperpolarizing factor (EDHF) [20]. Clearly, the above finding merits further studies.

Another possible and equally intriguing explanation is the blunting of the oxidative stress by valsartan. Oxidative stress affects NO bioactivity by reducing overall availability of locally released NO, both by accelerating NO deactivation and by reducing the disposal of essential eNOS precursors and cofactors such as BH4 and arginine [21]. In support of this mechanism, Hirooka and colleagues [22] have recently shown that valsartan therapy improved large-artery endothelial dysfunction by modulation of oxidative stress, evidenced as reduction of urinary excretion of metabolites directly involved in oxidative stress. In the same study, amlodipine had no appreciable effect on the markers of oxidative stress. Similarly, Aslam and colleagues [23] have shown that valsartan therapy significantly reduced plasma levels of asymmetric dimethylarginine (ADMA)—an endogenous inhibitor of NOS—and other plasma oxidative markers in hypertensive patients with end-stage renal disease.

Last, in our study, plasma levels of aldosterone, a mineralocorticoid, were significantly reduced by valsartan but not by amlodipine. Mineralcorticoids are known to enhance vascular responsiveness to pressor agents such as norepinephrine and angiotensin II even before systemic BP begins to increase [24,25]. In support of this, Taddei and colleagues [3] have reported that in patients with primary hyperaldosteronism, surgical excision of aldosterone-secreting adrenal adenomas normalized the vascular response to ACh, while the SNP response remained unchanged. It is possible that the significant reduction in plasma aldosterone achieved only during valsartan treatment might have been responsible for the improvement of both stimulated and basal NO release as compared with amlodipine treatment, despite the same blood pressure control achieved with both agents.

### Do Diverse Mechanisms of Endothelial Function Improvement Translate into Clinical Benefit?

Endothelial dysfunction has been shown to predict cardiac outcomes in hypertension. However, whether reversing this abnormality could have an impact on clinical benefit over and above that of BP and other risk factors has not been investigated. Nevertheless, reduction of vascular remodeling achieved through NO- dependent and -independent pathways may be an important goal to decrease cardiovascular risk, particularly in high-risk patients, such as those with metabolic syndrome or with clusters of cardiovascular risk factors, as found in patients with hypertension and diabetes. Perhaps, we could cautiously speculate that the significant reduction of developing diabetes mellitus in high-risk hypertensive patients during valsartan therapy recently seen in the Valsartan Antihypertensive Long-Term Use Evaluation (VALUE) study [26] might have been secondary to the favorable effects of valsartan exerted on vascular endothelium.

### Study Limitations

It could be argued that the difference in the results between amlodipine and valsartan might have been as a result of carryover effects. We think this is unlikely for two main reasons. First, BP after the second placebo period (washout) was similar in both treatment groups and was similar to that after the initial placebo run-in period (data not shown). Second, absolute FBFs between the two active treatment periods and placebo (baseline) were not different (Table 2).

## Conclusions

The present study found that valsartan, but not amlodipine, improved the functional properties of the resistance vessels in essential hypertension through a mechanism that was additional to that seen in BP reduction.

## Conflict of Interest

Thomas M. MacDonald has served on advisory boards for Novartis and was the UK coordinator for the VALUE study. Also, the same author has received research funding from Novartis to study the effects of valsartan in heart failure with normal left ventricular systolic function.
